# Reverse Shoulder Arthroplasty for Proximal Humerus Head-Split Fractures—A Retrospective Cohort Study

**DOI:** 10.3390/jcm11102835

**Published:** 2022-05-17

**Authors:** Jan-Philipp Imiolczyk, Ulrich Brunner, Tankred Imiolczyk, Florian Freislederer, David Endell, Markus Scheibel

**Affiliations:** 1 Center for Musculoskeletal Surgery, Charité-Universitaetsmedizin, 13353 Berlin, Germany; 2 Department of Trauma and Orthopedic Surgery, Krankenhaus Agatharied, 83734 Hausham, Germany; ulrich.brunner@khagatharied.de; 3 Department of Mathematics, University of Mannheim, 68131 Mannheim, Germany; imiolczyktankred@gmail.com; 4 Department of Shoulder and Elbow Surgery, Schulthess Clinic, 8008 Zurich, Switzerland; florian.freislederer@kws.ch (F.F.); david.endell@kws.ch (D.E.)

**Keywords:** head split, splitting, tuberosity, healing, union, trauma, humerus, trauma, low, high, energy, double shadow, pelican sign

## Abstract

Head-split fractures are proximal humerus fractures (PHF) that result from fracture lines traversing the articular surface. While head-split fractures are rare, surgical treatment of these complex injuries can be extremely challenging and is associated with high rates of complications. Treatment using primary reverse shoulder arthroplasty (RSA) has been associated with moderate complication rates and reproducible clinical results. The aim of this study was to evaluate clinical and radiographic outcomes, and complication rates of RSA for head-split PHF. Twenty-six patients were evaluated based on Constant Score (CS) and range of motion of both shoulders and Subjective Shoulder Value (SSV). Radiographic analysis evaluated tuberosity healing, prosthetic loosening and scapular notching. Patients achieved good clinical results with a CS of 73.7 points and SSV of 82% after a mean follow-up of 50 months. The relative CS comparing operated versus the unaffected shoulder was 92%. Greater tuberosity healing was achieved in 61%. Patients who suffered a high-energy trauma reached a significantly greater functional outcome. Patients who suffered multifragmentation to the humeral head performed the worst. There were no cases of loosening; scapular notching was visible in two cases. The complication rate was 8%. RSA is an adequate treatment option with for head-split PHF in elderly patients.

## 1. Introduction

Proximal humerus fractures (PHF) account for approximately 6% of all fractures [1]. The so-called head-split fracture describes a rare phenomenon (accounting for less than 5% of all PHF) that results from fracture lines traversing the articular surface of the humeral head [2]. This occurs when the impaction force of trauma acts in a vertical direction against the glenoid or acromion, such that shearing forces lead to humeral head cleavage. 

This type of fracture was originally diagnosed by a double shadow visible on plain anteroposterior (AP) radiographs, although it was usually regarded as a subtype of posterior dislocation fractures because of its rare occurrence [3,4,5]. Furthermore, the double shadow sign was easily missed on plain AP views of three patients in the first consecutive series including eight PHF patients [4]. Chesser et al. recommended the need for additional axillary radiographs and computed tomography (CT) scans to thoroughly diagnose these rare yet devastating fractures, which require early treatment to restore shoulder function [6]. If the pelican sign is detected on axillary views, a type II head-split fracture is diagnosed. The first arc represents the lesser tuberosity and the second arc a part of the articular surface which remained attached to the lesser tuberosity [2,7]. 

Therefore, a most recent attempt has been made to classify these specific fractures on CT scans by Scheibel et al. [7]. With the extension of CT for diagnosis or surgery planning, the event of fracture lines through the articular face can be diagnosed far better than on two-dimensional radiographs. 

Head-split PHF were first described in young male patients with high-energy trauma (i.e., a bicycle, motor or car accident) or epileptic seizures, where open reduction and internal fixation (ORIF) was considered the adequate treatment solution whenever closed reduction was not possible [6]. While these patients usually have good bone quality and the best potential for revascularization, it is important that these fractures are surgically fixed early after trauma in order to lower the risk of avascular necrosis and potential cartilage and joint degeneration [5,6,8]. Head-split fractures have also been reported in older, mainly female, patients involved in low-energy trauma (i.e., a simple fall from height) who typically have poorer bone quality and limited regenerative potential [2,7]. Conservative treatment for these particular fractures that are often misdiagnosed on plain radiographs has shown unsatisfactory results; in this instance, hemiarthroplasty (HA) was considered as a salvage procedure [6]. 

Reverse shoulder arthroplasty (RSA) has proven a reliable treatment option for severely displaced three- or four-part PHF in the older population, which offers encouraging mid-term results regarding pain loss, good return in range of motion and good functional outcome [9,10,11,12,13,14]. While ORIF and HA are both associated with high rates of complications (50% and 100%, respectively), and often followed by consecutive revision surgery, RSA may present as a potential treatment option even for relatively young patients aged below 70 years [15].

Given the sparse knowledge on the ideal treatment for this particular PHF and the accompanying high complication rates after HA and ORIF, the aim of this study was to evaluate clinical and radiological results as well as occurrence of complications in a unique consecutive series of head-split PHF patients treated with RSA.

## 2. Materials and Methods

### 2.1. Study Population

Between December 2009 and September 2020, 45 consecutive patients (m = 10, f = 35, mean age 75.8; range: 56–92 years at time of surgery) were identified with a head-split PHF. Of 45, 5 had sustained an additional glenoid rim fracture. All patients underwent RSA at one of two hospitals by one of three specialized shoulder surgeons. The indication for RSA was an unreconstructable PHF in an elderly patient population. All patients were retrospectively recruited via telephone invitation to attend a clinical follow-up examination. When patients could not be reached because the original contact details were no longer valid, we used the emergency contact details from medical records to gain further information on the patient’s current location and ensure follow-up assessment of these cases. For those patients unable to attend the clinical assessment because of age, poor health and/or the inability to travel to one of our clinics, we evaluated shoulder function and status only via telephone and postal contact. 

### 2.2. Implant Description, Surgical Procedure and Postoperative Rehabilitation Protocol

**Figure 1:**
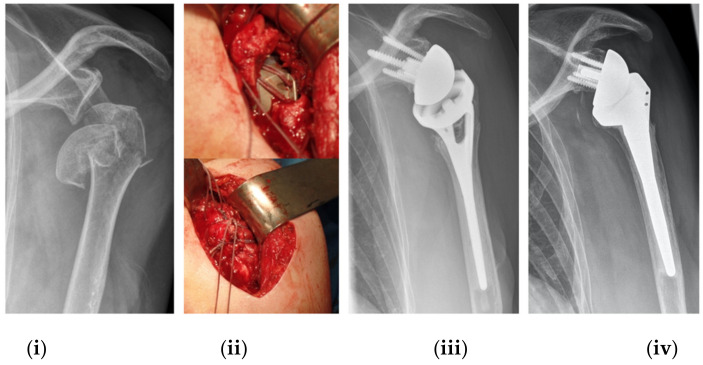
This figure shows a severe head-split PHF that has been treated with a RSA (**i**). All patients have received the same surgical treatment with a tuberosity refixation (**ii**). One year postoperatively, the greater tuberosity shows complete consolidation (**iii**). The *Fracture* stem (**iii**) shows a metaphyseal window to encourage bone ingrowth, whereas the *Reverse II* (**iv**) displays two holes for suturing the tuberosities. After 7 years of follow-up in another patient, however, the greater tuberosity has resorbed completely (**iv**).

Patients were treated in a beach chair position using a deltopectoral approach. For all RSA patients, a Grammont type of prosthesis (155° humeral inclination) was used with either a conventional (*n* = 5) or fracture-specific stem design (*n* = 40) and open metaphysis to allow bone ingrowth (AEQUALIS™ REVERSED II or AEQUALIS™ REVERSED FX, Tornier/Stryker Inc., Kalamazoo, MI, USA) (Figure 1). In addition, we applied a hybrid cementing technique to enable bone ingrowth at the metaphysis. In each case, both the greater and lesser tuberosities were anatomically reattached using FiberWire^®^ #5 sutures (Arthrex Inc., Naples, FL, USA) against the fin of the metaphyseal neck of the prosthesis as previously described [14]. After surgery, the shoulder was immobilized in a sling for 14 days. Passive mobilization began on postoperative day 15 and active mobilization was undertaken six weeks post-RSA. All patients completed the same standardized rehabilitation protocol.

### 2.3. Clinical Assessment

Patients were questioned about their history of trauma (low or high energy). In addition, the absolute as well as age- and gender-modified Constant-Murley score (CS), American Shoulder and Elbow Surgeons Assessment Form (ASES) score, Subjective Shoulder Value (SSV), Simple Shoulder Test (SST) and Activities of Daily Living requiring active External Rotation (ADLER) score were evaluated [16,17,18,19,20,21]. Abduction strength was measured using an Isobex 3.0 dynamometer (Veribor, Germany); pain was assessed using a scale of 0 to 15 points (15 = no pain; 0 = excruciating pain) and patient satisfaction (1 = unsatisfied; 2 = somewhat satisfied; 3 = satisfied; 4 = very satisfied) was also evaluated at the final follow-up examination.

Active range of motion (ROM), including anterior forward elevation, abduction, internal and external rotation, the Hornblower and external rotation lag signs (ERLS) were documented. Furthermore, ROM and CS were determined for the contralateral shoulder to assess the outcome of relative CS (i.e., the ratio of absolute CS of the affected versus contralateral shoulders).

### 2.4. Radiographic Evaluation

**Figure 2:**
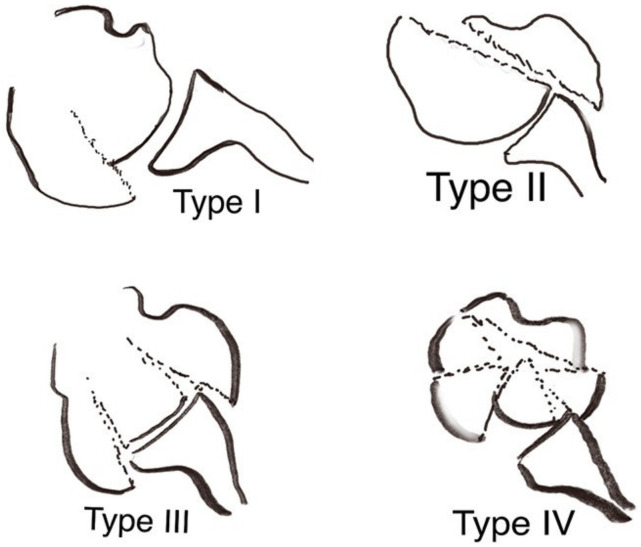
Four different types of head-split fracture patterns depending on involvement of the head-split component adjacent tuberosity (**Type I**: greater tuberosity; **Type II**: lesser tuberosity), whether fracture fragments split into disconnecting pieces that may lead to a stamp-like fracture pattern resulting in both greater and lesser tuberosity fragments connected to the articular face (**Type III**) or the multifragmentation of the disconnection of split pieces (**Type IV**) [7]. (Reproduced, with modification, under Creative Commons Attribution 4.0 International. License [https://creativecommons.org/licenses/by/4.0/ (accessed on 1 January 2022)], from: [7].

Preoperative radiographic assessments were made on standardized true AP, axillary and Y-view images. A CT scan was also performed to classify the head-split fracture type [7] and determine the presence of any additional glenoid rim fractures. The arrangement of patterns depends on the involvement of the head-split component adjacent tuberosity (Figure 2).

Postoperative evaluations were also made on true AP, axillary and Y-view radiographs to identify osteolysis, prosthetic loosening, heterotopic ossification and calcification, and scapular notching as well as tuberosity healing or migration. All radiographic examinations were evaluated by two independent surgeons, one of which was not involved in the surgical procedure.

### 2.5. Complication

All patients’ medical records were scanned for any shoulder-related complication or reoperation until the final follow-up examination.

### 2.6. Statistical Analysis

Due to the nature of this retrospective cohort study without a control group, all data are presented using standard descriptive statistics. Due to the small sample size, we applied the Wilcoxon rank-sum test to compare the clinical function of tuberosity healing, the presence of the ER lag sign and the difference in outcome after fracture pattern types I and IV. Analysis of variance (ANOVA) testing was applied to investigate any clinical differences among the various fracture types. Due to the small and nonrepresentational sample sizes for fracture types II and III (*n* = 3 each), we have used ANOVA testing just for investigation of tendencies. In addition, we also compared the outcomes of the “classic” type I fracture to those with comminution (type IV) to achieve a more reliable statistical analysis.

All statistical analyses were completed using SPSS 27 (IBM, Armonk, NY, USA) and the significance level was set to 0.05.

## 3. Results

Twenty-six patients (20 females, 6 males) with a mean age of 73.4 years were available for clinical and radiographic follow-up. The average postoperative follow-up occurred at 50 months (range: 12–142 months). A total of 19 patients dropped out of the study either because of death (*n* = 11), multimorbidity (*n* = 2) or severe dementia (*n* = 3) that hindered adequate examination of shoulder function; one patient suffered from paralysis following a severe stroke (*n* = 1), while two more dropped out either because of a SARS-COVID-19 infection (*n* = 1) or they were lost to follow-up (*n* = 1). There was no radiographic follow-up available for 3 of the 26 patients. Fracture morphologies and patient demographics are displayed in Table 1.

### 3.1. Clinical Results

**Figure 3:**
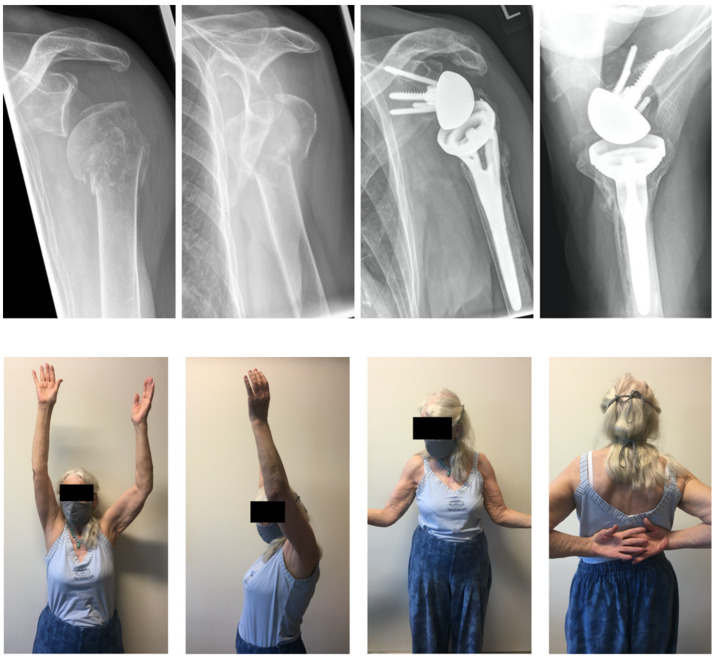
This 77-year-old woman sustained a type III fracture in preoperative radiographs (**upper two left**) after a fall onto her left shoulder while hiking. At 50 months post-RSA, the patient was very satisfied with excellent function (**lower bottom**) and a CS of 81 points, a relative CS of 99%, and a SSV of 95%. Both the greater and lesser tuberosities show healing and no scapular notching is visible on post-op (**upper two right**) images.

Our patient cohort achieved excellent clinical results based on all measured shoulder function scores and ROM (Table 2, Figure 3). The average SSV was 82% (range: 50–100%) and the absolute CS was 80% (range: 58–97 points). Most patients were pain free and reached full points in ADLER score and satisfaction. Patients in our cohort reached on average a flexion of 148° (range 100–175), external rotation of 15° (range: −10–60) and internal rotation up to L3 vertebra (range: thigh–scapula). Compared to the healthy, unaffected shoulder, the RSA shoulder reached 92% (range: 67–141%) of the contralateral function on average.

Patients who suffered a high-energy trauma performed significantly better and showed a greater absolute CS (79 vs. 69 points; *p* = 0.010), ASES (94 vs. 84 points; *p* = 0.044), ADLER score (30 vs. 26 points; *p* < 0.001) and SST score (87 vs. 68 points; *p* = 0.005) as well as abduction strength (5.0 vs. 2.9 kg; *p* = 0.005). With regard to ROM, patients after high-energy trauma reached greater anterior forward elevation (157° vs. 141°; *p* = 0.011), abduction (151° vs. 139°; *p* = 0.042) and external rotation (19° vs. 12°; *p* = 0.047) at final follow-up.

A total of seven patients presented with a positive ERLS, two of which also presented with a positive Hornblower sign. The presence of a positive ERLS coincided with significantly worse outcomes for SSV (72% vs. 84%; *p* = 0.023), the ADLER score (29 vs. 24 points; *p* = 0.002) and external rotation (1° vs. 21°; *p*= 0.002). Healing of both the greater (*p* = 0.02) and lesser tuberosities (*p* = 0.004) was observed when the ERLS was absent. 

### 3.2. Radiographic Results

There were no cases of osteolysis or prosthetic loosening at final follow-up in all patients with radiographic follow-up (*n* = 23). There were two patients with calcification of the posterosuperior cuff and another with heterotopic ossification around both the scapular neck and humerus. Scapular notching (Grade 1) was documented in two patients (8.7%). 

Greater tuberosity (GT) healing was present in 14 patients (61%) and the GT migrated superiorly and presented as a nonunion in five patients (22%). Four patients exhibited GT resorption at final follow-up (17%). Greater tuberosity healing was associated with greater ER (*p* = 0.03). Healing of the lesser tuberosity (LT) occurred in 18 patients (78%). There was no impact of LT healing on any clinical outcome measurement. However, higher rates of tuberosity healing were documented after a high-energy trauma.

### 3.3. Comparison of Head-Split Types

There were some differences in the absolute and modified CS, ASES score and internal rotation among the four fracture patterns (Table 3). Type 1 fractures seem to present the most favorable outcome, whereas fractures patterns with impairment of the LT (Type II–IV) seem to perform worse. In particular, LT head-split fragmentation seems to impact internal rotation the most.

When comparing the “classic” fracture type I with the GT adjacent head-split fragment to that with a comminuted articular face, type IV exhibited worse function in all clinical outcome measurements, particularly for internal rotation (*p* < 0.001). Although the GT healing rate was higher for type IV fractures, this did not reach statistical significance.

### 3.4. Complications

Of 26 patients, we reported a total of two (8%) complications throughout the post-RSA follow-up, one (4%) of which required revision surgery.

The first reported complication involved one patient who encountered instability 7 weeks post-RSA while undergoing physiotherapy. During a reoperation, the inlay liner was exchanged and a larger one was implanted. At 43 months after the secondary intervention, no further dislocations were encountered and a CS of 76 points and SSV of 70% was documented. The second complication was an acromion stress fracture reported at 39 months post-RSA. The affected patient declined the possibility of undergoing a reoperation after being informed of the potential risks associated with further surgery and opted for conservative treatment. This patient achieved the worst clinical outcome in our cohort with a CS of 52 points and forward flexion of 80°, but remained satisfied with their outcome despite a SSV of 70%.

After further investigations into our medical records, an additional patient was documented with a posterior dislocation 6 weeks post-RSA and underwent early revision surgery to increase the inlay liner. This patient was excluded from this analysis based on the diagnosis of severe dementia and inability to comply with the required follow-up examination.

## 4. Discussion

Patients with RSA after head-split PHF showed very good clinical and radiographic results, and the revision rate was 4% for those patients with clinical follow-up. Our cohort shows that patients who sustained a head-split fractures resulting from high-impact trauma had better results regardless of tuberosity healing. In addition, patients with a classic type 1 GT adjacent head-split showed better outcomes over those with multifragmentation of the articular face.

A current meta-analysis including 1303 PHF patients found the average anterior forward elevation flexion of about 122° with an average CS of 59 points and a total complication rate of 11% [22]. In our population, we achieved a similar complication rate, but our patients scored 74 points, on average, for the CS and achieved 148° elevation.

Healing of the GT led to favorable external rotation. Although GT healing was achieved in 61% of our cohort, this factor did not influence anterior forward elevation or any other functional outcome parameter besides external rotation, in contrast to a current meta-analysis [23]. This result could be biased by the small sample size in our cohort. However, the presence of the ER lag sign seems to be a prognostic factor not only for poorer external rotation but for subjective performance (SSV) since external rotation is involved in many activities of daily life.

Since our study population is older than 50 years, this study presents the advantages of RSA treatment for older patients. While complication rates for joint-preserving therapy options are high, young patients should be treated as soon as possible to minimize the risk of avascular necrosis [8,15]. High-impact trauma resulted in humeral head splitting that was first documented in dislocation fractures [4,6,8,24], yet we observed a collateral glenoid rim fracture in 5 out of 45 patients. We hypothesize that this is due to the trauma mechanism of the humeral head being forced against the glenoid, which causes the head-split fracture but may also result in glenoid rim fractures due to either extremely high shearing forces or poor bone quality.

The first published consecutive cohort included eight patients (3x ORIF, 3x missed, 1x CRIF, 1x HA): the oldest patient was a 56-year-old female who sustained a low-energy trauma fracture that was initially missed on radiographic examination and left untreated [6]. The outcome was a stiff and painful shoulder with extremely poor function. Conversely, younger patients (19- and a 21-year-old males) within the cohort who both suffered high-energy traumas achieved excellent functional results after early open or closed reduction and internal fixation (CS was 89 and 100 points, respectively) [6,8].

Although our study population is older by far, this finding concurs with our data including patients who had experienced a high-energy trauma and achieved a better outcome post-RSA. We hypothesize that head-split fractures resulting from high-impact and low-impact injuries are two different entities. Active patients who are confident to cycle or ski regularly can anticipate an increased risk of experiencing a high-impact accident. These patients could be considered biologically young as their active lifestyle results in good bone quality according to Wolff’s law [25]. In such cases, high shearing forces result in head-splitting fractures, but due to the great regenerative potential of vital tissue, patients can achieve better outcomes after RSA. Patients that sustain head-split fractures due to a fall from standing height were, on average, ten years older in our cohort and were not participating in an active lifestyle. For these cases, the fracture patterns are the result of poor bone quality and poor bone density due to immobility or osteoporosis.

Based on our cohort, articular-faced comminution of the humerus presents a serious treatment challenge for surgeons because very poor postoperative outcome can be expected. Our type IV patients had significantly poorer outcomes in all clinical scores measured. In addition, abduction and internal rotation were significantly lower for type IV fractures; external rotation was not affected by fracture type.

Although HA offers comparable results for head-split fractures (diagnosed on radiographs) compared to conventional three- or four-part PHF at short- to long-term follow-up [2,26], the complication rate of 36% and a revision rate of 12.5% should not be underestimated [2]. Compared to the cohort that has been treated with HA (*n* = 8), short-term results are comparable even though patients with RSA perform better in flexion but worse in external rotation [2]. Nowadays, RSA has limited the use of HA for PHF due to the current development and progress in shoulder arthroplasty [27,28]. 

Our study has several limitations, such as the retrospective design of this study as well as its small cohort. Differences between the different fracture types should not be considered as significant results; rather as trends. Our cohort analysis showed that head-split fracture patients were quite old with many in their mid-70s at the time of surgery, which resulted in a high rate of loss to follow-up due to death alone (24%). A strength of this study was that all patients were treated by only one of two senior surgeons in the same operative technique and that the head-split fracture was diagnosed on CT scans.

Finally, while joint-preserving therapy is the precedent for young patients with unreconstructable PHF, the high complication rates of 44% in cases aged under 55 years and up to 50% in general dictate the greater likelihood for secondary surgery due to osteonecrosis or nonunion [15,29]. As RSA techniques develop and push the boundaries of age due to good results in complicated fracture situations, long-term studies must continue to monitor whether young patients benefit more from early primary or later secondary RSA treatment.

## 5. Conclusions

RSA is a very good and reliable treatment option with low complication rates for proximal humerus head-split fractures in the older patient population. Patients who sustain a head-split fracture due to high-impact trauma have greater biological and regenerative potential that can lead to more promising outcomes. Comminution or multifragmentation of the articular face presents as a prognostic indicator for significantly poorer outcome. 

## Figures and Tables

**Table 1:** Baseline patient demographics.

N (women in %)	26 (77%)	
Age at surgery (years) (mean ± SD)	73.4 ± 7.8	
(range)	56–91	
Follow-up period (months) (mean ± SD)	50 ± 22	
(range)	12–142	
Trauma mechanism	Low energy	High energy
N	13 (50%)	13 (50%)
(Women in %)	85%	69%
Age at surgery (years) (mean ± SD)	77.8 ± 7.8	68.5 ± 7.3
(range)	(64–91)	(56–78)
Follow-up period (months) (mean ± SD)	52.6 ± 36.7	46.8 ± 22.0
(range)	(14–142)	(12–93)
Head-split classification * (*n*)		
I	3	7
II	3	0
III	2	1
IV	5	5
Additional glenoid rim fracture (*n*)	2	0

SD—standard deviation. * according to Scheibel et al. [7].

**Table 2:** Final postoperative clinical scores and range of motion.

	Mean (SD)	Range
Absolute CS (points)	73.7 (11.2)	43–92
Absolute CS of opposite shoulder (points)	80.3 (10.4)	58–97
Relative CS compared to opposite shoulder (%)	92.4 (14.1)	67–141
Age- and gender-modified CS (%)	79.1 (10.0)	53–95
ASES score (points)	89.1 (13.8)	53–100
SSV (%)	82.0 (13.0)	50–100
SST (%)	77.3 (19.4)	33–100
ADLER score (0–30 points)	27.7 (4.0)	12–30
Pain scale (0–15 points)	14.3 (2.0)	8–15
Abduction strength (kg)	4.0 (1.9)	0–8.7
Range of motion		
Anterior forward elevation (°)	148 (25)	100–175
Abduction (°)	144 (27)	80–180
External rotation in 0° abduction (°)	15 (16)	−10–60
Internal rotation (CS points)	6.1 (2.7)	0–10
Satisfaction (1–4)	3.8 (0.4)	3–4

SD—standard deviation; CS—constant score; ASES—American shoulder and elbow surgeons assessment form; SSV—subjective shoulder score; SST—Simple Shoulder Test; ADLER—activities of daily living requiring active external rotation.

**Table 3:** Clinical scores with regard to head-split fracture pattern types.

c	Type 1	Type 2	Type 3	Type 4	*p*-Value *	*p*-Value ** Type 1 vs. 4
	Mean (SD) Range					
*n*	10	3	3	10		
Age at surgery	77 (6.1)	76 (7.8)	71 (7.9)	69 (10.7)		
	68–87	70–85	62–77	56–91		
Follow-up period	43 (25.4)	80 (55.0)	47 (12.8)	49 (27.3)		
	14–95	37–142	33–58	12–93		
High-energy (*n*)/low-energy trauma setting (*n*)	7/3	0/3	1/2	5/5		
Age and gender modified CS (%)	87 (4.8)	73 (11.8)	78 (10.1)	74 (9.3)	0.010	>0.001
	80–95	60–81	67–86	53–86		
Absolute CS (points)	81 (6.3)	68 (10.0)	75 (7.8)	68 (12.7)	0.033	0.006
	72–92	57–75	66–81	43–84		
Relative CS to opposite shoulder (%)	100 (15.0)	82 (8.1)	90 (12.8)	89 (13.0)	0.2	0.06
	91–141	73–88	75–99	67–112		
ASES score (points)	98 (1.7)	86 (16.4)	77 (20.4)	85 (14.2)	0.047	0.002
	88–100	68–100	62–100	53–98		
SSV (%)	88 (9.5)	78 (25.7)	73 (19)	80 (9.1)	0.3	0.03
	70–100	50–100	60–95	70–90		
SST (%)	90 (12.3)	72 (21.0)	72 (17.5)	68 (20.6)	0.2	0.1
	67–100	50–92	58–92	33–92		
ADLER score (0–30 points)	29 (1.7)	24 (10.4)	25 (3.8)	28 (2.2)	0.053	0.050
	26–30	12–30	21–28	24–30		
Anterior forward elevation (°)	154 (23)	142 (28)	162 (3)	140 (28)	0.5	0.1
	110–170	110–160	160–165	100–175		
Abduction (°)	155 (22)	150 (17)	157 (23)	128 (29)	0.1	0.04
	120–180	130–160	130–170	80–165		
External rotation in 0° abduction (°)	16 (21)	13 (12)	10 (15)	16 (14)	0.9	0.4
	0–60	0–20	−5–25	100–175		
Internal rotation (CS points)	8.2 (1.4)	3.3 (2.3)	6.7 (2.3)	4.6 (2.7)	0.002	<0.001
	6–10	2–6	4–8	0–8		
Abduction strength (kg)	4.9 (1.9)	2.7 (1.0)	3.9 (0.8)	3.5 (2.2)	0.3	0.08
	2.5–8.7	1.5–3.4	3.3–4.8	0–7.2		
GT healing	4 out of 8 (50%)	1 out of 3 (33%)	2 out of 3 (67%)	7 out of 9 (78%)	0.5	0.3
LT healing	7 out of 8 (88%)	2 out of 3 (67%)	2 out of 3 (67%)	7 out of 9 (78%)	0.9	0.3
Scapular notching	0%	1 × Grade 1 (33%)	0%	1 × Grade 1 (11%)	0.02	0.5

SD—standard deviation; CS—constant score; ASES—American shoulder and elbow surgeons assessment form; SST – Simple Shoulder Test; SSV—subjective shoulder score; ADLER—activities of daily living requiring active external rotation; GT—greater tuberosity; LT—lesser tuberosity. * ANOVA for comparison of all four fracture types. ** Wilcoxon rank-sum test.

## Data Availability

Data available on request due to ethical restrictions. The data presented in this study are available on request from the corresponding author. The data are not publicly available.
